# Microbial resistance or team resistance?

**DOI:** 10.1186/1753-6561-5-S6-P285

**Published:** 2011-06-29

**Authors:** CN Shimura, J Da Silva, NEPECISS

**Affiliations:** 1 Escola de Edfermagem de Ribeirão Preto - USP, Ribeirão Preto, Brazil

## Introduction / objectives

Errors in the process of drug therapy has been an issue for the change in the epidemiological profile of microorganisms, causing antibiotic resistance. Whereas full-time care of the nursing staff in patient care, was aimed to analyze the errors which undoubtedly may be preventable with measures of interventions and reflect on nursing performance.

## Methods

The study is an integrative review in which 13 were selected articles concerning this topic through the electronic databases SciELO, MEDLINE and LILACS.

## Results

Studies show that bacterial resistance is increasing in parallel with antibiotic use, it is estimated by the World Health Organization (WHO) that 25 to 33% of patients use antimicrobial drugs during their hospitalization. Although the choice of antibiotic is a doctor's concern , the administration of drugs is carried out by nursing staff who are nursing assistants and technicians being supervised by nurses.

## Conclusion

The process is simple but common errors found in the studies were: patient error, error in dosage, route error, unauthorized drug error and error of time being the most prevalent. The misuse of such substances affect the microbial ecology, diminishes the possibilities for therapeutic treatment of infectious processes, generate long hospitalizations and expenditures. The quality of nursing practice is impaired by long working hours, shifts, reduced staff, many functions and services, yet the nurses must strive and offer safe practice. The knowledge of the drug and its use in surveillance, recognition of error, and improvement courses are essential to prevent medication errors and therefore minimize microbial resistance. There is a paradigm extensive to be addressed since most errors are preventable.

## Disclosure of interest

None declared.

